# The Occurrence, Uses, Biosynthetic Pathway, and Biotechnological Production of Plumbagin, a Potent Antitumor Naphthoquinone

**DOI:** 10.3390/molecules30071618

**Published:** 2025-04-04

**Authors:** Polavarapu B. Kavi Kishor, Bangaru Naidu Thaddi, Rajasheker Guddimalli, Tukaram Dayaram Nikam, Krothapalli Raja Surya Sambasiva Rao, Rupasree Mukhopadhyay, Prashant Singam

**Affiliations:** 1 Department of Genetics, Osmania University, Hyderabad 500007, India; rajguddimalli@yahoo.in (R.G.); prashantsingam@gmail.com (P.S.); 2 Department of Life Sciences, Aditya Degree & P.G. College (Autonomous), Kakinada 533003, India; dr.thaddi_bn@aditya.ac.in; 3 Department of Botany, Savitribai Phule Pune University, Pune 411007, India; tdnikam@unipune.ac.in; 4 Department of Pharmacy, Mangalayatan University-Jabalpur, Jabalpur 483001, India; krssrao@yahoo.com; 5 Department of Genetics & Biotechnology, Veeranari Chakali Ilamma Women’s University, Hyderabad 500095, India; rupasree.ucw@gmail.com

**Keywords:** anticancer compound, bioreactors, cell cultures, hairy roots, plumbagin

## Abstract

Plumbagin is an important naphthoquinone with potent anticancer properties besides multitudinous uses in healthcare. It is produced in a limited number of species and families but mostly in the roots of Plumbaginaceae family members. The biosynthetic pathway and the genes that regulate plumbagin synthesis are not completely known, but details of these are being revealed. Several species, including *Plumbago*, *Drosera*, and others, are being uprooted for the extraction of plumbagin by pharmaceutical industries, leading to the destruction of natural habitats. The pharmaceutical industry is therefore facing an acute shortage of plant material. This necessitates enhancing the accumulation of plumbagin using suspensions and hairy roots to meet market demands. Many factors, such as the aggregate size of the inoculum, stability of the culture, and the sequential effects of elicitors, immobilization, and permeabilization, have been demonstrated to act synergistically and markedly augment plumbagin accumulation. Hairy root cultures can be used for the large-scale production, growth, and plumbagin accumulation, and the exploration of their efficacy is now imperative. The secretion of compounds into the spent medium and their in situ adsorption via resin has remarkable potential, but this has not been thoroughly exploited. Improvements in the quality of biomass, selection of cell lines, and production of plumbagin in bioreactors have thus far been sporadic, and these parameters need to be further exploited. In this review, we report the advances made relating to the importance of stable cell line selection for the accumulation of compounds in long-term cultures, hairy root cultures for the accumulation of plumbagin, and its semicontinuous production via total cell recycling in different types of bioreactors. Such advances might pave the way for industrial exploitation. The steps in the biosynthetic pathway that are currently understood might also aid us in isolating the relevant genes in order to examine the effects of their overexpression or heterologous downregulation or to edit the genome using CRISPR-Cas9 technology in order to enhance the accumulation of plumbagin. Its potential as an anticancer molecule and its mode of action have been amply demonstrated, but plumbagin has not been exploited in clinics due to its insolubility in water and its highly lipophilic nature. Plumbagin-loaded nanoemulsions, plumbagin–silver, or albumin nanoparticle formulations can overcome these problems relating to its solubility and are currently being tried to improve its bioavailability and antiproliferative activities, as discussed in the current paper.

## 1. Introduction

Plants are a rich source of chemical diversity that can be used in traditional as well as modern medicine [1,2,3]. The global trade for herbal medicine was valued at USD 216.40 billion in the year 2023 (https://www.fortunebusinessinsights.com/herbal-medicine-market-106320, accessed on 28 December 2024), is increasing at a rate of 7%, and is anticipated to reach USD 5 trillion by 2050 (Government of India, 2000) [4]. The utilization of herbal medicine directly affected 4 billion individuals worldwide in the year 2002 (WHO, Geneva 2002) [5]. Chinese traditional medicine is used by nearly 1.5 billion people across the world to promote health and wellness [6]. Between the years 2005 and 2007, 13 plant-derived natural products were approved as drugs [7]. Secondary plant products include a whole gamut of natural products such as alkaloids, anthraquinones, flavones, flavonoids, glycosides, pigments, terpenes, and others [3]. Phytochemicals, which have the potential to cure many diseases, are used extensively in sectors like the cosmetics, nutraceutical, and pharmaceuticals industries, including homeopathy [8]. Such bioactive compounds can help in combating insect pests that destroy plant species, and they may be produced in extremely minute quantities [9]. Secondary metabolites have complex stereostructures with many chiral centers, which may be essential for their biological activity. This complexity makes them unique, and hence, it is extremely difficult to chemically synthesize such bioactive compounds on a commercial scale [1,10]. Medicinal plants are being exploited ruthlessly throughout the world, resulting in the depletion of natural resources and genetic stock [11]. Under these conditions, it is necessary to adapt biotechnological methods for the large-scale production of bioactive compounds. The following section discusses the occurrence of plumbagin in diverse families and genera.

## 2. Plant Families That Synthesize Plumbagin

Several families biosynthesize 1,4-naphthoquinones, including Ancistrocladaceae, Avicenniaceae, Balsminaceae, Bignoniaceae, Boraginaceae, Dioncophyllaceae, Droseraceae, Ebenaceae, Gentianaceae, Juglandaceae, Iridaceae, Plumbaginacea, Scropulariaceae, and Verbenaceae [12]. Some algae, fungi, and bacteria, as well as a few animals, also accumulate plumbagin [12]. Several species produce medicinally important plumbagin (5-hydroxy-2-methyl-1,4-naphthoquinone) in the range of 0.15 to 1.34 g/dry weight (g/dry wt) of roots; these include *Aristea*, *Aldrovanda*, *Ancistrocladus*, *Ceratostigma*, *Dionaea*, *Dioncophyllum*, *Diospyros*, *Drosera*, *Drosophyllum*, *Juglans*, several *Plumbago* species, and *Triphyophyllum*, belonging to the families Ancistrocladaceae, Dioncophyllaceae, Droseraceae, Drosophyllaceae, Ebenaceae, Juglandaceae, Iridaceae, Nepenthaceae, and Plumbaginaceae [13,14,15,16,17,18,19,20]. The currently available literature shows that members of the Plumbaginaceae are the primary sources of plumbagin, and these are being exploited in industry and by traditional medical practitioners. *Plumbago rosea* synonymous *P. indica*, synonymous *P. coccinea*, synonymous *Thela coccinea* is an evergreen, perennial herb or shrub [21]. *Plumbago* species grow very slowly, reach up to 1.5 m tall, are usually branched and belong to the class Magnoliopsida, division Magnoliophyta and family Plumbaginaceae [22,23]. It is commonly called Indian leadwort, and it is grown as an ornamental plant in the tropics. In Ayurveda, it is named as chitramula and chitrak and grown for its brilliant inflorescence (Chitrak: https://www.planetayurveda.com, 2019, accessed on 14 November 2024) [24,25]. The plant produces deep pink or scarlet flowers, which mostly takes place in winter. *Plumbago rosea* is native to southeast Asia, Indonesia, Philippines, southern parts of China, and Sri Lanka. It is widely distributed throughout India [22,26,27]. Plumbagin has been detected in both Ancistrocladaceae and Dioncophyllaceae family members [28]. Plumbagin is a yellow-colored naphthoquinone pigment [29,30]. It is soluble in acetone, alcohol, benzene, and chloroform [31]. Although a variety of plants accumulate plumbagin, it is the members of Plumbaginaceae that have been studied extensively. Accordingly, this review focuses on the accumulation of plumbagin mostly from *Plumbago* species.

In vitro studies indicate that naphthoquinones such as plumbagin and anthraquinones accumulate in callus, suspensions, adventitious and hairy roots [32,33,34]. Roy and Bharadvaja [35] reviewed the process of plumbagin extraction, isolation, estimation and its pharmaceutical activities. But a comprehensive review covering different aspects of in vitro plumbagin production from diverse taxa has been lacking. For commercialization, stable cell lines with high productivity are indispensable. Furthermore, the optimization of growth parameters for good biomass production coupled with a high accumulation of bioactive compounds either in the cells or spent medium is crucial. Thus far, the efforts have been limited to callus and shake flask cultures and the standardization of techniques for the elicitation of the compound plumbagin using a limited number of elicitors. Therefore, there is a compelling need to enhance the accumulation by techniques such as permeabilization and immobilization along with in situ adsorption. Such attempts were highly scattered or sporadic, especially in *Plumbago*. But for upscaling the suspension cultures or hairy roots for industrial-scale production, either the suspensions or the hairy roots must be grown in suitable bioreactors with necessary engineering considerations in long-term cultures without any loss of cell viability and decline in secondary metabolite accumulation. Studies on plumbagin accumulation in *Plumbago* species are available, but reports on the scale-up of plumbagin in cell cultures are limited [36,37,38]. The review by Pandey et al. [39] does not comprehensively deal with the effect of cell aggregate sizes and the importance of selection of superior cell lines with a high accumulation or stable cell lines that synthesize plumbagin in long-term cultures, in situ adsorption and the production of plumbagin in large-scale bioreactors. In this review, we make an effort to bring out the assorted uses of plumbagin in detail, the steps known thus far in the biosynthetic pathway, the current status of enhanced plumbagin production in callus, suspensions, and hairy root cultures, and the need for deploying genetic engineering or genome editing technologies so as to adopt such methods for industrial-scale production. The impact of co-cultivation, elicitation, immobilization, permeabilization, their synergistic effects and the significance of the in situ adsorption of bioactive compounds that can assist in the large-scale production and high biomass yield in different bioreactors have also been discussed in this review with our insights.

## 3. Detection of Plumbagin in Diverse Taxa

*Ancistrocladus heyneanus* (family Ancistrocladaceae) has been reported to accumulate plumbagin [40]. The genus *Diospyros* belongs to the family Ebenaceae, and several species of *Diospyros* are used extensively for treating many diseases such as chest pains, epilepsy, gonorrhea, malaria, measles, psychiatric disorders, snake bites, sterility, and others in traditional systems of medicine [41,42]. While plumbagin has been detected in roots of *Diospyros kaki* [43], in *D. shimbaensis*, plumbagin as well as 3,3′-biplumbagin have been reported [42]. Among the members of the Droseraceae, *Drosera burmanii*, *D. capensis*, *D. indica*, *D. uniflora* and *Dionaea muscipula* have been reported to produce plumbagin [44,45,46,47,48,49]. The in vitro production of plumbagin from *Drosera* species has been reported first by Finnie and van Staden [50]. The in vitro detection of plumbagin from *Drosera adelae*, *D. aliciae*, *D. binata*, *D. burmannii*, *D. capensis*, *D. cuneifolia*, *D. gigantean*, *D. intermedia*, *D. magna*, *D. muscipula*, *D. peltata* Smith var. lunata, and *D. ramentacea* has been reported [15,45,47,51,52,53,54,55,56]. *Drosphyllum* belongs to the family Drosophyllaceae, which also accumulates plumbagin. Species that accumulate plumbagin in this family include *Drosophyllum natalensis* and *Drosophyllum lusitanicum* [57,58]. *Juglans regia* belongs to the family Juglandaceae, and plumbagin has been detected in the husks of the walnuts [59]. In *Juglans cinerea*, *J. nigra*, and *J. regia*, plumbagin accumulation has been detected as well [59,60]. In the families Ebenaceae, Drosophyllaceae, Droseraceae, and Juglandaceae, the concentration of plumbagin generally ranges between 0.0004 and 0.242 g/dry weight in vivo but not in vitro [40,42,55,56,57,58,59]. Further, these families have limited distribution in different geographical locations. Hence, pharmaceutical industries do not extract the plumbagin from these families, since it is not economically feasible. The plant *Nepenthes* belongs to the family Nepenthaceae. Members of the genus *Nepenthes* such as *N. alata*, *N. fusca*, *N. gracilis*, *N. khasiana*, *N. mirabilis*, *N. superba*, *N. thorelli*, *Nepenthes thorellii* × (*ventricosa* × *maxima*), *N.* × *ventrata* (*N. alata* × *N. ventricosa*), and *N. ventricosa* synthesize plumbagin [17,61,62,63,64,65,66,67]. Plumbagin was detected in the roots (1.33% on dry weight basis) of *N. khasiana* [68]. Plumbagin has also been identified in *Nepenthes* × *ventrata* [66]. *Nepenthes* again is a carnivorous plant with regional distribution and limited availability for the pharmaceutical industries. In the family plumbaginaceae, plumbagin has been detected from species such as *Plumbago angustifolia*, *P. auriculata*, *P. capensis*, *P. europaea*, *P. rosea* (also called as *P. indica*), *P. scandens*, *P. zelanica* and others. *Triphyophyllum peltatum* belongs to the family Dioncophyllacae, which is a part-time carnivorous plant. It becomes carnivore when it does not obtain nutrients from the soil, and plumbagin has been detected in this species [69]. In addition to the above, from *Lawsonia inermis* (family Lytheraceae), isoplumbagin has been reported [70]. It is the members of the Plumbaginaceae family that have cosmopolitan distribution (from arctic to tropical regions) with a relatively higher accumulation of plumbagin in comparison with other families. Hence, *Plumbago* species are collected by uprooting them with a consequence of the large-scale destruction of their habitat. This warrants the adaptation of biotechnological methods for the in vitro production of plumbagin.

## 4. Plumbagin in Human Healthcare

Plumbagin has assorted uses in human healthcare. Its chemical and pharmaceutical aspects have been reviewed by Badwaik et al. [18]. Plumbagin exhibits biological activities such as antioxidant, anti-inflammatory, antibacterial, antifungal, anti-atherosclerosis, antileishmanicidal (used for killing insects), analgesic and antidiabetic [71,72,73,74,75,76,77,78,79,80]. The following are some of the therapeutic activities of plumbagin.

### 4.1. Anticancer Activity

Plumbagin is an analog of vitamin K3 and a prooxidant [81]. The mechanism of action of plumbagin in tumor suppression [82] and the cytotoxic potential of the compound for cancer therapy have been explored [83]. Plumbagin targets antiangiogenesis, apoptosis, autophagy, and antimetastasis pathways in addition to cell cycle arrest [81]. Plumbagin shows potential against diverse types of malignant tumors and has become a hotspot of research [81,83,84,85,86,87]. Sandur et al. [60] demonstrated that plumbagin regulates cellular proliferation by activating the nuclear factor kappa B (NF-κB) pathway. Plumbagin inhibited the growth of breast cancer cells but not the normal breast epithelial cells [88]. Plumbagin has been demonstrated to induce apoptosis in Her2-overexpressing breast cancer cells via a mitochondrial-mediated pathway [89]. Whole plant extracts of *P. zeylanica* showed anticancer activity against MCF-7 and HT-29 cell lines [90]. Plumbagin has been found to decrease cancer cell survival by inducing cell cycle arrest and mitochondria-mediated apoptosis [91,92]. In human breast cancer cells, plumbagin caused apoptosis, inhibited the telomerase activity and arrested the G2/M cell cycle [93]. It appears that plumbagin disrupts sulfhydryl homeostasis and proteasomal function [93]. Ethanolic extracts of *P. zeylanica* also work well against Dalton’s ascitic lymphoma in mice [94]. The anticancer activity of plumbagin has been tested against brain tumor, breast cancer, canine cancer, cholangiocarcinoma, esophageal cancer, hepatocellular carcinoma, leukemia, lung cancer, melanoma, osteocarcinoma, prostate cancer, and squamous carcinoma [83,93,95,96,97].

### 4.2. Mode of Action of Plumbagin Against Cancer Cells

Plumbagin modulates the apoptotic pathway, cell cycle regulation pathway, inflammatory pathway, ROS induction pathway, and signal transduction pathway like P13K/AKT/mTOR, STAT3/PLK1/AKT and others [98]. As a prooxidant, plumbagin induces reactive oxygen species (ROS), suppresses glutathione, and causes DNA double-strand break by oxidative DNA base damage [99,100]. Plumbagin suppresses the invasion and migration of breast as well as gastric cancer cells by inhibiting the expression of chemokine receptor CXCR4 [101]. The proliferation of cancer cells is suppressed by plumbagin due to the inhibition of angiogenesis through the Ras signaling pathway and stimulation of vascular endothelial growth factor (VEGF) receptor-2 [102]. Plumbagin modulates *nuclear factor kappa B* (*NF-κB*), *signal transducer and activator of transcription 3* (*STAT3*), and *Ak strain transforming* (*AKT*) genes. It represses proliferative and inflammatory responses of T cells independent of ROS generation by modulating intracellular thiols [103]. Also, Wang et al. [104] reported that plumbagin suppresses the lipopolysachharde (LPS)-induced inflammation by inactivating *NF-κB* and mitogen-activated protein kinase (MAPK) signaling pathways in RAW 264.7 cells [104]. Further, it has been noticed that plumbagin suppresses the proliferation and survival of esophageal cancer cells and human bladder cancer cells via STAT3-PLK1-AKT and P13K/AKT/mTOR signaling pathways and epithelial–mesenchymal transition (EMT), respectively [98,105,106]. Other important mechanisms of plumbagin-suppressed cancer cells include dihydroorotase (DHOase), which is a key enzyme in the de novo biosynthesis of pyrimidine nucleotides and a target for chemotherapy. Guan et al. [107] noticed that plumbagin is a competitive inhibitor of DHOase. Furthermore, the binding of plumbagin to DHOase occurs through the loop-in mode, which may serve as a drug target [107]. The discovery of new targets like DHOase may open the doors for effective chemotherapy. When treated with plumbagin and xanthohumol combination, the survival of mouse models affected by pancreatic cancer has been found to be enhanced spectacularly. It appears, therefore, that plumbagin serves as a potential new treatment with copper and also xanthohumol combinations. The copper–plumbagin complex suppressed the proliferation of human cervical carcinoma, human breast cancer cell lines, and murine melanoma (B16F10) with reasonably good IC50 values. Interestingly, this complex displayed stronger toxicity against breast carcinoma and skin melanoma cells in comparison with that of non-cancerous cells [108]. Such a display of specific toxicity only toward cancer cells and antiproliferative activity by plumbagin makes it a promising and a potent antitumor molecule. Mukherjee et al. [108] reported that the Cu–plumbagin complex depolymerizes microtubules and triggers ROS and the damage of DNA specifically in cancer cells. Plant-based bioactive compounds generally deploy their effects through copper, which generates ROS, thereby initiating apoptosis in cancer cells. El Oridi [109] demonstrated that plumbagin hampers the growth of pancreatic cancer cells PNAC-1 and MIA PaCa-2 by availing the copper present in the cells. The cell death caused by plumbagin has been found to be decreased by the copper chelator neocuproine. But plumbagin prevents copper from leaving cancer cells by inhibiting the expression of *CTR1* and *ATP7A* genes. Both these proteins stimulate copper uptake in cells [110,111,112]. If the copper transporters are silenced, H6c7 cells become sensitive to plumbagin in the copper-enriched medium, demonstrating that the interaction of plumbagin with cellular copper is critically important for growth inhibition against cancer cell lines. Notably, the findings from the experiments carried out by El Oridi [109] infer that plumbagin targets the copper metal present in the nucleus and subsequently triggers oxidative stress, accelerating cell death. The discovery also brings out the new role and the potential of plumbagin as a key therapeutic agent to counter the proliferation of cancer cells by the copper-dependent copper-redox cycle mechanism. Such innovative strategies help us to combat cancer effectively and efficiently. Furthermore, a combination of plumbagin and xanthohumol synergistically work against multiple human pancreatic cancer cell lines [108,113]. The combination alters the levels of B-cell lymphoma (BCL2), which is connected with apoptosis. By distilling the aforementioned findings, and from the available literature [83,114], it is evident that plumbagin causes cancer cell apoptosis by effectively mobilizing the copper ions bound mostly to chromatin, which eventually trigger copper-redox reactions and generate ROS, leading to the oxidative DNA damage of cancer cells [114,115]. The mechanism of action of plumbagin therefore appears certainly distinct from the rest.

### 4.3. Plumbagin and Its Ability to Re-Sensitize Chemo and Radiation-Resistant Cancer Cells

One of the restraints in cancer therapy is malignant cells becoming invulnerable to both chemo and radiotherapies [99]. Such resistance is due to a pro-survival MAPK/Extracellular Signal-Regulated Kinase (ERK) pathway machinery operative in cancer cells [116]. After activation, the ERK protein kinases phosphorylate the modulators of apoptosis, which lead to the proliferation of cells again [117]. Curiously, when used alone or in combination, plumbagin displays its remarkable ability to re-sensitize chemo and radio-resistant cancer cells, which has been amply proved [83,98,118]. Paclitaxel-induced cell death has been enhanced by plumbagin when added in combination [119]. Plumbagin overcomes paclitaxel resistance in breast cancer cells through ERK-regulated apoptosis induction [119]. Giacomini et al. [120] showed that in A431/Pt-resistant cancer cells (cisplatin-resistant cells), plumbagin stimulates G_2_/M phase cell cycle inhibition and enhances the cell apoptosis again. In line with this, plumbagin has been noticed to diminish the hyperphosphorylated form of the retinoblastoma protein, thus bringing about the suppression of the retinoblastoma complex, which confirms the arrest of the cell cycle in cancer cells resistant to cisplatin therapy [120].

### 4.4. Anticoagulation, and Cardiovascular Diseases

Plumbagin’s structure is akin to vitamin K. Low doses of plumbagin (2 mg/kg body weight) when administered to animals prolonged the bleeding time by altering platelet adhesiveness and coagulation [121]. Such an anticoagulation activity might be helpful to modulate cardiovascular diseases.

### 4.5. Antidiarrheal Activity

Plumbagin suppresses activities of the calcium-activated chloride channel (CaCC) and cystic fibrosis transmembrane conductance regulator (CFTR) channels, thereby helping to prevent secretory diarrhea in HT-29 cells and mouse colons [122].

### 4.6. Antifertility

Edwin et al. [123] studied the effect of *P. zeylanica* leaf extracts on the estrous cycle of rats. They noticed the antifertility activity of female albino rats. Vishnukanta and Rana [124] evaluated the antifertility activity of the leaves of *P. zeylanica* in immature ovariectomized female Wistar rats. Extracts displayed antiestrogenic activity and caused changes in the functions of the uterus.

### 4.7. Anti-Inflammatory

Plumbagin reduced the levels of glutathione (GSH) with a concomitant rise in the reactive oxygen species (ROS). It appears that thiol groups but not ROS play crucial roles in the plumbagin activity. Plumbagin abolished the mitogen-induced phosphorylation of ERK, IKK and degradation of IκB-α but not P38, JNK and AKT. The results indicate that the antiproliferative effects of plumbagin are mediated by the regulation of redox and the application of thiol-depleting agents as probable anti-inflammatory drugs [103]. Plumbagin as a vitamin K3 analogue abrogates lipopolysaccharide-induced oxidative stress, inflammation and endotoxic shock through NF-κB suppression [125]. The potential anti-inflammatory activity of *P. zeylanica* was reported by Subramaniyan and Paramasivam [126]. They noticed that this activity is due to its radical scavenging properties. Poosarla [127] showed that plant extract can be used for treatment of arthritis using an adjuvant induced arthritic rat model. Plumbagin also has been found to ameliorate the hepatic ischemia–reperfusion injury in rats. Its role of high mobility group box 1 in inflammation, oxidative stress and apoptosis has been extensively illustrated [128].

### 4.8. Antimalarial

Plumbagin has been tested against malarial parasites in vitro as well as in animal models. Pradeepa et al. [129] detected mosquito repellent activity against the malarial vector. Concentrations that inhibit 50% of the growth of *Plasmodium falciparum* parasites (IC50) have been studied by Sumsakul et al. [130]. Plumbagin at a dose of 25 mg/kg body weight administered for 4 days exhibited mild antimalarial activity [130].

### 4.9. Antimicrobial and Antibiofilm Activities

Importantly, plumbagin exhibits remarkable activity against bacteria and fungi. While root extracts of *P. zeylanica* displayed activity against *Staphylococcus aureus* [131], alcoholic extracts inhibited the growth of *Shigella* and *E. coli* [132] as well as *Salmonella typhi* and *Staphylococcus aureus* [133]. Similarly, leaf extracts also showed inhibitory effects against *Bacillus cereus* and *Candida* [134]. It inhibits quorum sensing-modulated virulence and biofilms of Gram-negative bacteria [135]. It is amazing to note that plumbagin resurrects colistin (an antibiotic medication)-susceptible bacteria (*Pseudomonas aeruginosa*) which are otherwise resistant against colistin [136]. Further, it shows antifungal and antibiofilm activities against *Cryptococcus neoformans* [137]. It increases antimicrobial and antibiofilm capacities against *Klebsiella pneumonia*. At the same time, it diminishes the resistance mutations [138].

### 4.10. Antimutagenic

Studies show that plumbagin is not a mutagenic in the stationary phase of cell growth, but it is slightly mutagenic in the exponential phase in *Escherichia coli* AQ634 cells. Plumbagin exhibits antimutagenicity to mutagenicities induced by 2-nitrofluorene (2NF) and 1-nitropyrene in *Salmonella typhimurium* [139]. But at higher concentrations, plumbagin displays cytotoxic effects [140].

### 4.11. Antinephrotic

Alcoholic extracts of *P. zeylanica* displayed a nephroprotective effect in cisplatin-induced nephrotoxicity in Swiss albino mice [141].

### 4.12. Antiobesity and Antidiabetic

Extracts of *P. zeylanica* when administered to humans showed significant weight loss [142]. In diabetic rats, the extract of the same plants enhanced the hexokinase and decreased the glucose-6-phosphatase activities simultaneously and thus helped in combating diabetes [143]. Plumbagin reduced the blood glucose levels and plasma insulin [74]. The root bark of *P. zeylanica* is used for controlling obesity [27]. Thus, plumbagin has manifold functions, and it is being used amply in the traditional medicinal system in India. However, whether plumbagin alone causes these effects or a combination of bioactive molecules is thus far obscure.

### 4.13. Antirheumatic

Roots of *P. zeylanica* have been used for treating rheumatism, laryngitis, diseases of the spleen, and decoction of seeds against muscular pains [17,144].

### 4.14. Aphrodisiac

Leaves of *P. zeylanica* exhibit aphrodisiac properties [27,145,146].

### 4.15. Arteriosclerosis and Cough

The extracts of *Drosera* species are used for treating arteriosclerosis, cough, inflammation, and syphilitic infection [50].

### 4.16. Digestive Problems, Piles, and Liver Disorders

Traditionally, *P. zeylanica* is used for treating piles, colitis, ascites, and liver disorders [146]. In the Ayurvedic system of medicine, flowers are used for easy digestion.

### 4.17. Neuroprotective

Plumbagin acts as a neuroprotective agent. Such an activity against intracerebroventricular (ICV) lipopolysaccharide (LPS)-induced behavioral deficiencies has been discovered in rats. The results indicate that the neuroprotective activity of plumbagin is moderated by the alleviation of oxidative stress and inflammation [147].

### 4.18. Limitations of Plumbagin for Use in the Clinics and the Ways to Improve Its Bioavailability

Plumbagin is only slightly soluble in water, weakly acidic in nature, and highly lipophilic with delayed absorption, a short residence time of 5 h, and 38.7% bioavailability in rats [118,148]. Because of these characteristics, plumbagin has not yet been tried in clinics. However, efforts have been made to enhance its bioavailability using nanoemulsions and nano-based delivery systems such as liposomes (tiny lipid particles), niosomes (lamellar molecules) and micelles (aggregate of molecules in a colloidal solution). Chrastina et al. [149] used plumbagin-loaded nanoemulsion-based formulations (1–3.5% of surfactants) and their impact on prostate cancer cells (PTEN-P2). Nanoemulsions displayed improved antiproliferative activity in comparison with that of plumbagin controls [149], inferring that nanoemulsion formulation is a better delivery system. Kamble et al. [150] noticed an improved bioavailability of plumbagin with a self-nanoemulsifying drug delivery system. These findings indicate that nanoemulsions improve not only the antiproliferative activity and efficacy of plumbagin but also its bioavailability. However, clinical trials are highly crucial to make plumbagin a potent therapeutic agent for cancer intervention.

## 5. Elucidation of Biosynthetic Pathway of Plumbagin

**Figure 1:**
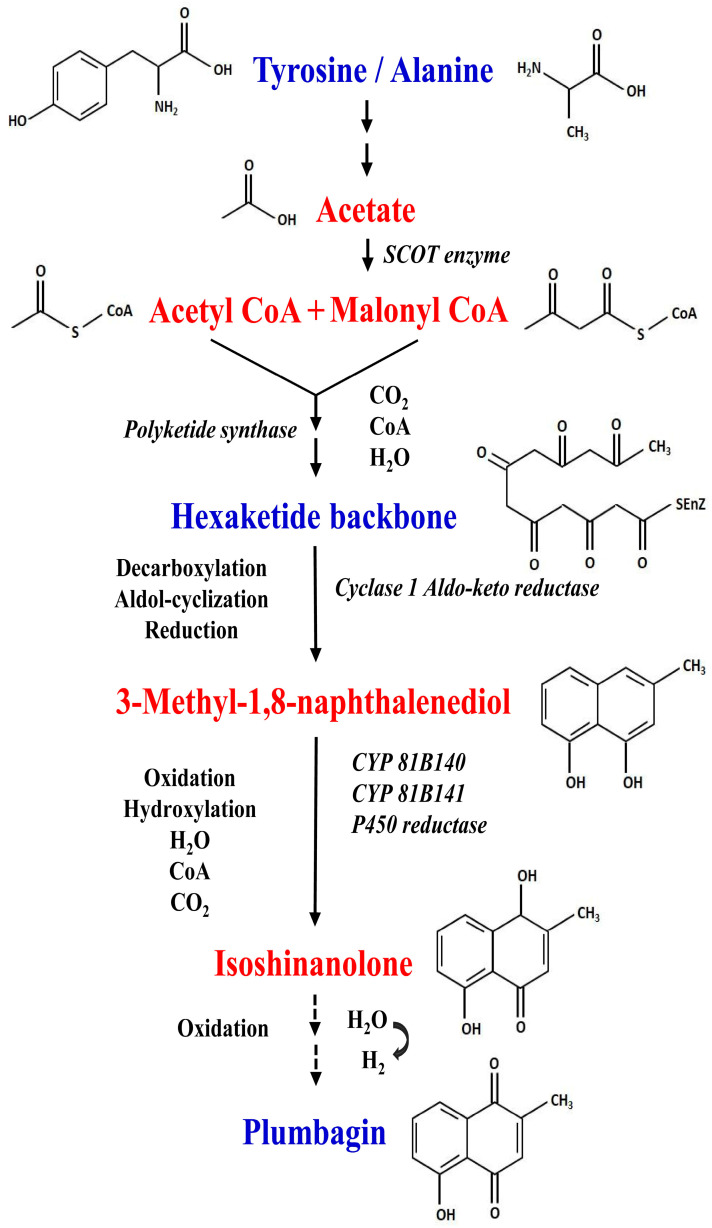
The figure displays the putative biosynthetic pathway of plumbagin. (SCOT = succinyl-CoA: 3-ketoacid CoA transferase; CYP = cytochromes, CO_2_ = carbon dioxide, CoA = coenzyme A). Pathway is not completely illustrated, and all the corresponding genes have not been revealed. While the aromatic amino acid tyrosine appears as the remote precursor for the biosynthesis of plumbagin, isoshinanoline is the immediate precursor. Dotted lines indicate that the exact steps and enzymes for the conversion of isoshinanolone to plumbagin are unclear. However, oxidation reaction occurs in the final step of the pathway. Alanine, another amino acid (not shown in the pathway), may also participate in some plants in which plumbagin biosynthesis occurs.

Labeled experiments revealed that shikimate-7-C,L-^14^CH_3_-methionine, DL-tyrosine-β-^14^C, DL-phenylalanine (ring-1-^14^C), and DL-mevalonic acid-5-^14^C have not been incorporated into plumbagin. On the contrary, acetate-1-^14^C, 2-^14^C, and malonate-2-^14^C-labeled compounds have been incorporated into plumbagin, indicating that the polyacetate–malonate pathway is the route for its biosynthesis [151]. Bringmann et al. [40] investigated the plumbagin pathway by feeding ^13^C_2_-acetate and later by ^13^C NMR analysis to the suspension cultures of *Ancistrocladus heyneanus*. They elucidated the acetogenic origin and polyketide folding mode of plumbagin in the biogenesis, indicating that it originates from acetate. In contrast, labeled alanine fed to the *Nepenthes insignis* pitchers has been incorporated into the plumbagin but not into other glycoside compounds like plumbaside A. This indicates that *Nepenthes* uses the C2 portion of the carbon skeleton of alanine [152]. Furthermore, labeled sodium acetate has not been incorporated into any of the secondary metabolites, inferring that alanine acts as a precursor for plumbagin biosynthesis in *N. insignis* [152]. Plumbagin is produced via the acetate and polymalonate pathways also in many plants [153,154]. These differing statements conclude that different biosynthetic pathways (alanine or acetate–malonate pathways) might be used for plumbagin biosynthesis in diverse taxa. Tyrosine is the precursor that is converted to homogentisate, acetate and then to plumbagin through a series of reactions [151,155] (Figure 1). Its biosynthesis involves aldol condensation, aldol cyclization, dehydration, hydration, hydroxylation and oxidation reactions. Analysis of the transcriptome and metabolome revealed few key enzymes and genes implicated in the plumbagin biosynthetic pathway. Among them, *aldoketoreductase*, *polyketide cyclase*, *CYP81B140* and *CYP81B141* genes have been found to be important in *P. zeylanica* [155]. In this species, one molecule of acetyl coenzyme A (Co-A) and five molecules of malonyl Co-A combine to form hexaketide backbone, which is catalyzed by the enzyme polyketide synthase. The hexaketide backbone then undergoes decarboxylation, aldol-cyclization and reduction reactions to form 3-methyl-1,8-naphthalenediol by two enzymes namely *Pz*cyclase 1 and *Pz*aldo-keto reductase 1. The compound 3-methyl-1,8-naphthalenediol is converted to the precursor of plumbagin isoshinanolone via oxidation and hydroxylation (Figure 1). Isoshinanolone then is converted to plumbagin by oxidation, after which it is catalyzed by the cytochromes *Pz*CYP81B140, *Pz*CYP81B141 and *Pz*P450 (Figure 1) [155]. Li et al. [20] studied the effect of methyl jasmonate (MeJA) on hairy root cultures of *P. auriculata* and found a dramatic accumulation of plumbagin through the induction of the jasmonic acid (JA) signaling, shikimic acid and methylvaleric acid (MVA) pathways. They performed a global analysis of the hairy root cultures of *P. auriculata* by RNA-seq profiling which showed a high expression of phenylalanine ammonia lyase 3 (PAL3) and 3-hydroxy-3-methylglutaryl CoA reductase (HMGR). These two enzymes activated other genes such as *chalcone synthase* (*CHS*), *isopentenyl diphosphate* (*IPP*), and *farnesyl pyrophosphate synthase* (*FPS*) in the pathway. The stimulation of these genes has resulted in the enhanced synthesis of plumbagin. The authors postulate that aldehydes, ketones, and polyketones accumulate due to the stimulation of these genes [20]. The results point out that the methylvaleric acid pathway is vital for plumbagin biosynthesis. Further, using molecular dynamic simulations, Muralidharan et al. [156] unraveled an important enzyme namely naphthoate synthase, which catalyzes the cyclization of *O*-malonyl benzoyl CoA to an unknown intermediate with two possible structures. This intermediate is again catalyzed by thioesterase enzyme to produce plumbagin, but these in silico studies do not completely unravel the pathway [156].

## 6. Need to Explore Biotechnological Methods to Produce Plumbagin

*Plumbago* species grow slowly and take several years to accumulate plumbagin and to produce quality roots [38]. Plumbagin is produced predominantly in the epidermal cells and cortex of roots; hence, whole plants are generally uprooted to extract the bioactive compound. In the years 2004 to 2006, the demand for the roots in India was ~1285 tons, but it is estimated to increase by 10% every year [157,158]. The demand for plumbagin is escalating because of its potential pharmacological activities [159]. The supply of raw material is dwindling due to the destruction of the natural habitat [32,158,160,161]. The demand for the production of plumbagin is increasing from many pharmaceutical companies. This is coupled with low supply, meager yields and its overexploitation of natural resources [38,159,160]. This has sparked the need to look for strategies that can help to escalate the production of plumbagin without destroying the natural habitat [162]. Furthermore, the accumulation of plumbagin in the intact plants would vary depending upon the age, developmental stage, season, geographical location and extraction method used [163]. On the contrary, plant tissue, cell and hairy root cultures have the potential to produce bioactive compounds on a large scale and throughout the year irrespective of the season and country [164,165]. Furthermore, the genes involved in its biosynthesis and the biosynthetic pathway are not completely known for genetic engineering or genome editing. Epigenetic factors that modulate the plumbagin biosynthesis are also unknown so far. The chemical synthesis of plumbagin has been discovered [166], but chemical routes are highly carbon intensive. Synthetic biology helps us to produce secondary metabolites, but finding suitable host organisms and appropriate gene manipulations are not always available [167]. Since there is a need to curtail carbon emissions in the wake of climate change, one should fall back on cultured plant cells and hairy roots for the biosynthesis, since the natural biosynthetic routes are distinctly energy and carbon-efficient [168,169,170]. Under these sets of conditions, it is crucial for us to exploit biotechnological methods including genome editing technologies for the large-scale production of plumbagin. Using cell and organ cultures, shikonin, berberine, taxol, many cosmetics and others have been produced on an industrial scale [1,171,172,173,174]. Callus cultures have been exploited for the large-scale production of secondary plant products such as α-tocopherol, ajmaline, flavonoids, paclitaxel, reserpine, resveratrol, serpentine, etc. [175]. Many researchers opine now that callus/suspension and hairy root cultures are a reliable source for extracting therapeutic metabolites [11,176,177,178]. Suspensions can be generated, and also single cell clones from callus can be further exploited in bioreactors for commercial production [11,160,161,179,180,181]. Aside from callus and suspension cultures, shoots, adventitious and hairy roots have also been deployed for the production of pharmaceutically important compound accumulation in vitro [164,182,183,184,185,186]. Hairy roots are versatile tools for high productivity as well as for genetic stability in long-term cultures [165,187,188]. Therefore, the exploitation of suspensions and hairy roots for the commercial-scale production of pharmaceutically important compounds from rare, endangered and endemic plants needs expeditious exploration.

## 7. Induction of Callus and Suspensions from *Plumbago* Species and Plumbagin Accumulation

The initiation of callus and suspension cultures from different explants has been reported from *Drosophyllum lucitanicum* [189], *P. rosea* [190], *P. europaea* [38], and *P. zeylanicum* [35,191]. The production of bioactive compounds in callus and suspension cultures depends on the biomass accumulation. Generally, an inverse correlation exists between biomass build up and metabolite accumulation [192]. The influence of hormones and selection of stable cell lines of *P. rosea* for the accumulation of plumbagin has been worked out [160] (Table 1). Acetylsalicylic acid and ammonium-induced somatic embryogenesis have been reported from *Plumbago rosea* suspension cultures [193]. Komaraiah et al. [193] noticed significant boost in the accumulation of plumbagin from the suspensions and hairy roots. Hairy root cultures were reported from *P. rosea*, but suspensions, root and hairy root cultures have not been exploited further at the commercial level using bioreactors [194]. Silja and Satheeshkumar [195] reported the establishment of adventitious root cultures from leaf explants and improved plumbagin via elicitation in *P. rosea*. Jose et al. [196] reported on the in vitro cultivation of hairy roots of *P. rosea* in a reaction kettle. Roots were initiated from nodal explants of *P. zeylanica* on Murashige and Skoog’s (MS) medium [197] containing auxins. Root suspension cultures of *P. zelanica* were optimized for the increased accumulation of plumbagin [35]. Hairy root and adventitious root cultures from *P. zeylanica* have also been reported [198]. The initiation of callus and suspension cultures has been reported from *P. rosea* [160,161,190,199]. In general, callus initiation from leaf explants was good on MS medium fortified with 3% sucrose and 1 mg/L naphthaleneacetic acid (NAA) [194]. Plumbagin content ranged from 0.19 mg/g dry cell weight to 0.89 mg/g dry cell wt. Embryogenic callus cultures were also reported from *P. rosea* on MS medium supplemented with 1 mg/L indole-3-acetic acid (IAA) and 0.3 mg/L 6-benzylaminopurine (BAP). While embryogenic cultures accumulated 6–10 mg/g dry cell wt of plumbagin, non-embryogenic cultures accrued 2–4 mg/g dry cell wt of plumbagin [160,193,194]. These experiments indicate that differentiated cultures accumulate significantly higher concentrations of plumbagin in comparison with non-embryogenic cultures. Perhaps, cell–cell contact in somatic embryos or differentiated organs is vital for such a differential accumulation. However, our understanding at the molecular level about what makes embryogenic cultures synthesize higher amounts of secondary products is dismal.

Suspension cultures were initiated by inoculating the callus cultures into MS liquid medium enriched with 1 mg/L IAA, 0.5 mg/L NAA and 0.3 mg/L BAP. Suspensions were inoculated onto MS medium supplemented with 1 mg/L IAA, 0.5 mg/L NAA, and 0.3 mg/L BAP along with different concentrations of NH_4_ by adjusting the potassium nitrate concentration in the medium. Acetyl salicylic acid (ASA) (up to 2 mg/L) enhanced the number of somatic embryos formed per culture at 1.5 mg/L and 1 mg/L IAA [193]. The plumbagin content in suspensions increased gradually up to 4.3 mg/g dry cell wt by the 16th day in culture in *P. rosea*. Also, both growth and plumbagin content were optimal when the pH of the medium was around 5.2–6.0 [193,194]. Suspensions appear to synthesize and amass more plumbagin in comparison with callus cultures, although the reasons are precisely not known. However, agar–agar that is added into the medium for solidification or the chemicals present in it may inhibit the accumulation of secondary metabolites. The in vitro accumulation of plumbagin from diverse taxa under varying hormonal and elicitor concentrations is shown in Table 1.

## 8. Cell Aggregate Size Optimization as a Novel Method for Plumbagin Accumulation

The general tendency among the researchers working in the realms of tissue culture is to use callus or suspensions for the production of secondary plant products. Such routine experiments generally do not churn out copius amounts of bioactive compound accumulation in culture. Therefore, one has to adapt novel techniques such as the determination of optimal cell aggregate size for the sizeable production of bioactive compounds for subsequent exploitation by the pharmaceutical industries. Cell aggregate sizes as process variables have been involved in affecting the biomass and also bioactive molecules in culture. Cell aggregate size is a key parameter in determining the accumulation of secondary products. In suspensions of *P. rosea*, 4.3 mg/g dry cell wt/L was reported [190]. Cells in suspensions generally form aggregates. It appears that such cell aggregates influence both growth and metabolite synthesis. Cell aggregates measuring 500 µm in diameter displayed 8.3 g dry cell wt/L, while plumbagin accumulation reached 1.83 mg/g dry cell wt/L [190]. Plumbagin content was optimum (4.7 mg/g dry cell wt/L) at 1500 µm cell aggregate size, but with further increases in the size up to 2000 µm, the biomass accumulation decreased (6.5 g dry cell wt/L) (Table 1). The hypericin content in *Hypericum perforatum* suspensions enhanced with an increase in cell aggregate size up to 500 to 1000 µm [215]. Thus, it appears that a certain degree of cell aggregation is imperative for the optimum biosynthesis of secondary metabolites in culture. These results also point out the influence of adjacent cells on secondary metabolism. But whether it is the physiological gradients of materials that exist in cells or other factors that stimulate the synthesis is not clear yet. On the other hand, cell aggregates that are 500 µm in diameter stimulated the growth of cells in *Tinospora cordifolia* (9.6 g dry cell weight), but larger cell aggregates promoted 3.8 mg/g dry weight of tissue [216]. Smaller aggregates (690 µm) of *Taxux cuspidata* suspensions accumulated 22 mg/L paclitaxel than cultures with larger (1100 µm) cell aggregate sizes, which accumulated 11 mg/L [217]. Aggregate cells larger than 2 mm accumulated triptolide with optimum biomass. Smaller aggregates ranging from 0.5 to 2 mm in diameter accumulated higher amounts of triptolides when compared to larger cell aggregates in *Tripterygium wilfordii*. Contrary to this, cell aggregate sizes did not affect the contents of wilforgine and wilforine in suspension cultures of *T. wilfordii*. Noticeably, the smallest aggregates (0.1–0.5 mm) showed lower biomass with chloroplasts but higher alkaloid accumulation [218]. However, the factors that control the cell aggregate size are not completely known. Similarly, the genes and the epigenetics that regulate the cell aggregate size vis-à-vis the accumulation of bioactive molecules need to be thoroughly explored. It appears that arabinogalactan proteins are implicated in the cell aggregation of cell suspensions of *Beta vulgaris* [219]. Patil et al. [220] pointed out that cell aggregate size is important for the long-term viability of cells to produce paclitaxel in suspension cultures of *Taxus*. With the enhanced cell aggregate size, the content of flavonoids also increased in *Ficus deltoidea* [221]. Nonetheless, these experiments point out the importance of validating cell aggregate sizes as well as optimizing the same as a targeted process, since cell aggregate sizes undoubtedly play critical roles in determining the biosynthesis and accumulation of bioactive compounds in vitro.

## 9. Screening and Selection of a Large Number of Stable Cell Lines for Plumbagin Accumulation in Suspensions of *P. rosea*

The selection of cell lines that accumulate high levels of metabolites is a novel method. Callus and suspension cultures represent heterogeneous masses of cells, but each cell unveils varying biosynthetic potential for natural product accumulation. Largely, the biosynthesis and accumulation is organ and genotype specific. Screening begins with the selection of a suitable genotype and explant with inherent potential for the large-scale accumulation of bioactive compounds. Therefore, the selection of organs is critical for callus and suspension culture initiation [183,222]. Varying capabilities of cells to produce natural products also necessitate screening the cells for genetic variation to accumulate bioactive or pharmacologically important compounds [160,194]. Such natural variation for the biosynthesis of bioactive compounds has not received enough attention by the plant biologists, since the methods are often cumbersome, labor intensive and time consuming. Few attempts have, however, been made for the selection of cell lines with improved accumulation. One of the cell lines (PR10) selected from *P. rosea* suspensions produced 5.496 mg/g dry wt of plumbagin (Table 1). Cells often lose their ability to synthesize and accumulate natural products as the cell line effectively ages [160,172,194]. Such a decrease in the biosynthetic potential of the cells is due to genetic instability, the loss of desirable characteristics and somaclonal variations, or a combination of factors including mutations [163,190,222]. Chromosomal rearrangements and genetic modifications (deletions and insertions) occur during long-term culture including ploidy changes, and such a change in ploidy may affect the synthesis and accumulation of biomolecules [223]. In line with this, a varied DNA content (due to change in ploidy) in one-year-old cultures of *Taxus* has been noticed [224]. Higher ploidy also causes the silencing of multiple genes [223]. Genetic changes that occur in cultured cells lead to alterations in the accumulation of biomolecules, entailing the periodic screening of cells that maintain a high accumulation of bioactive molecules [224]. The implications of subpopulations in the accumulation of secondary products over a long period in culture and their behavior have received considerable interest. Zhang and John [225] demonstrated a faster growth of older cultures of *Nicotiana plumbaginifolia*. This phenomenon has been attributed to the increased levels of cyclin-dependent kinase activity, decreased cytoskeleton and loss of regeneration capacity after a prolonged culture of suspensions and also mutations in such cells. However, our understanding about the ploidy level and secondary metabolite accumulation in cultured cells is poor; hence, extensive research is needed in order to design a strategy for improving the accumulation despite variations in ploidy levels. Hence, as an alternative to callus and suspension cultures, hairy roots are preferred, since they are relatively genetically stable. Genes may be methylated or demethylated; histone modifications and small RNA-based mechanisms may occur in cultured cells, impacting the accumulation of secondary products. Also, epigenetic changes that may occur during the prolonged culture of cells in vitro may affect the pathways and accumulation of compounds. Cell culture aging has been linked to mutations that occur in cell cultures of *Coffea arabica* [226]. In long-term cell cultures of *Taxus media*, cv Hicksii DNA methylation has been detected which modulated the biosynthesis of paclitaxel [227]. Such epigenetic changes in cultured cells provide us with crucial clues for finding out the gradual loss of bioactive compound accumulation in long-term cultures. This implies that multilayered regulation occurs for the secondary metabolite accumulation in plants [228]. Furthermore, metabolic pathways involved in the biosynthesis of secondary metabolites are mostly compartmentalized in plastids, but plastid development in cultured cells may be incomplete, which may affect the biosynthesis profoundly [229].

To sidestep this, and for sustainable accumulation in cells, Raven et al. [230] used a selectable marker coupled with the flow sorting technique. Such a strategy resulted in a high accumulation of recombinant proteins in plant cells for more than one year in a bioreactor. Other factors that may contribute to such a loss are not yet known and need to be further explored. If cultures need to be exploited for the industrial production of compounds, it is indispensable to select cell lines that do not lose their ability to accumulate natural products over a long period of time. Such a screening of suspensions involves the isolation of individual cells or a few celled clumps and their culture for longer periods of time to test their efficacy. In *P. rosea*, a large degree of variation in biomass production and plumbagin accumulation was noticed, though the callus was derived from a single leaf [160,194]. Several cell lines were screened using cell aggregate cloning by visual and analytical methods [194]. It has been observed that biomass and plumbagin accumulation are negatively correlated. Komaraiah et al. [160] demonstrated that *P. rosea* (PR) cell lines PR8 and PR9 accumulated 12 to 15 g of biomass on dry cell wt/L, but plumbagin accumulated 1 to 2.5 mg/g dry cell wt. Interestingly, PR10 accumulated both biomass (5.634 g/dry cell wt/L) and 4.562 mg/g of plumbagin on a dry cell wt basis (Table 1). This line also displayed a higher accumulation of plumbagin consistently for more than one year with regular subcultures [160,194]. These studies point out that cell line selection is decisive for maintaining the high sustainability of both growth and secondary metabolites. Such a selection process can also be facilitated by the exogenous application of an intermediate molecule involved in the targeted pathway. Shoots of *Mentha arvensis* were screened in vitro for tolerance to menthol concentrations. Dhawan et al. [231] identified genotypes with a better ability to accumulate higher quantities of menthol. Such selections might help to produce the compounds on a commercial scale. Cell lines with a high accumulation of rosmarinic acid in *Lavendula vera* were obtained by adding m-phenylalanine and *p*-fluoro-DL-phenylalanine in the medium, which then trigger the activity of the phenylalanine ammonia-lyase (PAL) enzyme [232]. Cell lines that are tolerant to high amounts of the phenylalanine analogue produce elevated levels of rosmarinic acid but not the susceptible ones [232]. Largely, progress to improve the accumulation of bioactive molecules over longer periods has been slow. Our understanding of how to modulate the patterns of unstable secondary metabolite accumulation and produce consistently high levels has thus far been proven limited. The gradual loss of secondary metabolite accumulation is an established fact now [194,205,233]; therefore, future research studies should aim at overcoming some of these bottlenecks.

## 10. Source of Light, Precursor Feeding and Accumulation of Plumbagin in In Vitro Cultures of *Plumbago rosea*, *Drosera burmannii* and *D. indica*

The source of light and feeding the precursor to the growth medium significantly improve the accumulation of secondary metabolites in cultured cells, but they have not been widely tried in *Plumbago* species. In general, white-fluorescent light is used for stimulating the growth and production of secondary plant products in cultured cells or hairy roots [234]. The effect of artificial light emitting diodes (LEDs) on the accumulation of secondary products has been tried at specific wavelengths [234]. In *D. indica*, shoots accumulate approximately at 15 mg/g dry wt and roots 2 mg of plumbagin per g dry wt under blue LED light in cultures exposed for 14 days [49]. The results indicate that blue LED light is a good source for improving the plumbagin content in cultured cells. An improved accumulation of plumbagin was detected in in vitro grown *Dionaea muscipula* plants in response to the quality and quantity of light [235], suggesting that plumbagin is associated with photoprotection and antioxidant activity in *D. muscipula* plants. The above experiments amply demonstrate that light significantly improves the accumulation of plumbagin, indicating that light is necessary for plumbagin biosynthesis.

Acetate–mevalonate and alanine are both precursors for plumbagin biosynthesis [49]. In cultured cells, precursors have been fed to boost the metabolites in cell suspensions of *Achyranthes aspera* [235] and shoot cultures of *Ruta graveolens* [236]. Feeding 5 mM L-alanine to root cultures of *P. rosea* for 14 days coupled with the in situ adsorption of plumbagin with styrene–divinylbenzene resin (Diaion^®^ HP-20) enhanced the plumbagin content to 22.4 mg/g dry wt in comparison with untreated roots (1.6 mg/g dry wt). Feeding L-alanine alone devoid of the adsorbent resulted in 14.4 mg/g dry wt plumbagin accumulation in *P. rosea* [237]. In *Drosera indica*, the content of plumbagin has increased in shoots to 9.850 mg/g dry wt by feeding 50 mg/L of the precursor sodium acetate for three days. Shoots accumulated higher plumbagin when compared to roots in both *D. indica* and *D. burmannii*. Further, the content of plumbagin was higher in *D. indica* than in *D. burmannii* [49]. Tyrosine also acts as a precursor for plumbagin, but feeding experiments have not been carried out in vitro.

## 11. Feeding Conditioned Medium and Combination of Metabolic Modules

Cells in vivo accumulate secondary products, since they coordinate with other cells. Some metabolites may be synthesized in one tissue of an organ or cell organelle of a plant but the other molecule in another organ or cells. Such a metabolic activity or synergy may be lacking in cultured plant cells, since partitioning between different cell types is mostly deficient [238]. In tobacco, nicotine is synthesized in roots, which may act as a precursor for the biosynthesis of nornicotine—a compound likely to have potential for abuse that accumulates in leaves [238]. Such a metabolic partitioning is usually either absent or insufficient in cultured cells. But, conditioned medium if fed to the cultured cells, as a strategy to enhance the secondary metabolite accumulation, helps accumulate the required product. Rajabi et al. [239] have shown that the nornicotine production of NtomCYP82E4 cells can be triggered by feeding a conditioned medium from NtabMPO1 overexpression. They have coupled two different cell types (supernatant generated by a donor cell type and metabolically different receiver cell type) to enhance the metabolite content [239,240]. These experiments point out that the co-cultivation of cells that activate nornicotine/bioactive compound accumulation or diverse metabolic modules can promote the target compound synthesis. However, such experiments involving the addition of spent medium into the cultured cells and coupling two different cell types have not been often performed in medicinal plant cell cultures including *P. rosea*.

## 12. Elicitation of Callus and Suspension Cultures for Plumbagin Accumulation

Both biotic and abiotic elicitors have been found effective to augment the content of plumbagin in *P. indica*, but abiotic elicitors are more effective when compared to fungal (*Aspergillus niger* and *Rhizopus oryzae*), bacterial (*Bacillus subtilis* and *Pseudomonas aeruginosa*), and yeast extract [199]. The time of elicitation and concentration of the elicitor are vital to obtain the optimum accumulation of the bioactive compounds. Bhaskar et al. [241] noticed that biotic elicitors are a boon for the in vitro production of plant secondary metabolites. In majority of the publications, different elicitors with varying concentrations have been tried without optimizing the time of addition to the cultures. However, the time of addition of elicitor plays an important role in *P. rosea* [199]. Such an important observation helps us to experiment and augment the biosynthesis in cultured cells of *P. rosea*. The results show that the concentration of biotic/abiotic elicitors including chitosan and also the time of elicitation are crucial stimulants affecting the bioactive compound accumulation. It is desirable to harvest secondary metabolism without destroying the cells in culture. Therefore, attempts were made to collect plumbagin secreted into the spent medium. In addition to portraying as an elicitor, chitosan also acts as a permeabilizing agent. The addition of 150 mg/L chitosan results in a 70% release of plumbagin into the exterior of cells, but fungal elicitors tested on suspension cultures were less effective in mimicking an identical response [199]. Chitosan has induced the accumulation of plumbagin from suspension and root cultures of *P. rosea* [161,238] (Table 1). Chitosan upregulated the *phenylalanine ammonia lyase* (*PAL*) gene and enhanced the phenylpropanoid compounds in *Scrophularia striata* cells [242]. It appears therefore that chitosan triggers the biosynthetic pathway genes and thereby enhances the accumulation of plumbagin. Salicylic acid enhanced the plumbagin concentration in embryogenic suspensions of *P. rosea* [243]. The authors have also studied the effect of jasmonic acid, yeast extract and auxins as elicitors and found decreased culture viability but enhanced plumbagin accumulation (5.59-folds), probably through the stimulation of the pathway genes. The results indicate the need to generate viable cells for over the long term.

In callus cultures derived from the roots of *P. zeylanica*, yeast extract and salicylic acid improved the plumbagin yields by 6.5- and 3.4-fold, respectively, in comparison with those of the controls [212]. Salicylic acid has the ability to modulate the specific biosynthetic pathway genes either singly or in combination with other elicitors, owing to its hormonal activity. Further, salicylic acid is cost-effective and eco-friendly; hence, it can be tried in cultured cells for large-scale production [244]. Roy and Bhardvaja [35] reported the highest plumbagin in *P. zeylanica* by optimizing culture parameters in half-strength MS liquid medium supplemented with 3% sucrose and 2 g/L inoculum density. In *P. zeylanica*, yeast extract, malt extract, methyl jasmonate and salicylic acid significantly enhanced the plumbagin content [35]. Both salicylic acid and methyl jasmonate can bring about the stimulation of the pathway by kinases that transduce the signals in the downstream, produce ROS, and activate ion fluxes besides causing cytoplasmic acidification. These series of reactions activate the genes associated with the pathway, leading to a higher accumulation of the bioactive compound [245]. Bacterial lysate (elicitor) enhanced the plumbagin content by 2.6-fold in comparison with that of the control in *Drosera muscipula* and *D. capensis* [45]. Putalun et al. [47] and Gangopadhyay et al. [158] noticed such an enhanced production of plumbagin with the addition of elicitors in *D. burmannii* cultures. Yeast extract stimulated the accumulation of plumbagin by several folds in comparison with wild-type cells of *D. indica* [47,48]. The treatment of in vitro grown *D. indica* plantlets with chitosan and salicylic acid triggered plumbagin accumulation after 3 days of treatment [48]. The plumbagin content was 2.69 mg/g dry weight with yest extract as an elicitor in comparison with control plants (0.50 mg/g dry wt) [48]. High plumbagin content (3.45 mg/g dry weight) was recorded in shoot clumps of *D. peltata* in the presence of 2,4-dichlorophenoxyacetic acid (2,4-D) [211].

## 13. Elicitation in Adventitious Root Cultures

In addition to suspensions, root cultures have also been employed for plumbagin production [207]. Improved yields of plumbagin from root cultures of *P. indica* were reported by both biotic and abiotic elicitors, especially chitosan [246,247,248]. Root cultures from leaf explants of *P. rosea* were established, and the content of plumbagin has been found to be enhanced in cultures [195]. Some of these elicitors may create oxidative stress which releasees ROS. ROS, in turn, participate in biotic and abiotic stresses and trigger the secondary metabolism, being the signal transducers [249]. Feeding the cultures with L-alanine and in situ adsorption have been found to boost the plumbagin levels in *P. indica* root cultures [119,249]. Simultaneous heat shock and in situ adsorption also resulted in increased plumbagin production in *P. indica* root cultures [247]. Jaisi et al. [209] reported that gamma rays can enhance the plumbagin content in root cultures of *P. indica*. Gamma rays cause the genetic mutations, and hence, plumbagin content is likely to improve. In *P. zeylanica* cultures, the application of *Azospirillum* increased the plumbagin in roots up to 0.027% [250]. But, none of the above parameters were able to trigger significantly high levels that would be suitable for industrial-scale production. The method, however, points out that we need to explore the process of elicitation further in organ cultures of *P. rosea* for boosting the content of plumbagin.

## 14. Simultaneous and Sequential Dual Elicitations, an Innovative Approach

Elicitation triggers the upregulation of genes implicated in defense- and non-defense-related genes, besides phosphorylation and dephosphorylation events [251]. The recurrent usage of elicitators is common in cultured cells, but sequential dual elicitation is sparse. The use of such novel methods should be widespread so as to boost the production. The elicitation of hairy roots led to an enhanced accumulation of secondary metabolites, but if the product accumulates in the spent medium, product recovery becomes easier. Such an accumulation prevents the feedback inhibition of accumulated compounds. Its removal from the spent medium also prevents the degradation of the compound of interest [252,253,254]. Simultaneous and sequential dual elicitations in *P. indica* root cultures are novel, which inferred that a combination of 150 mg/L chitosan with 5 mM L-alanine or 2 mM methyl-β-cyclodextrin improves plumbagin production [255]. While chitosan + L-alanine addition to a 14-day-old culture resulted in 14.62 mg/g dry cell wt, the sequential addition of methyl-β-cyclodextrin to a 12-day-old culture followed by chitosan supplementation yielded 14.33 mg/g dry cell wt [255]. The above experiments indicate that sequential elicitations or simultaneous elicitation coupled with immobilization, immobilization treatment time, and duration of treatment need to be optimized for each culture and are indispensable to enhance the product. If the product is not removed from the spent medium, then the accumulation of the bioactive compound may be decreased due to feedback regulation. The above experiments apparently indicate that metabolite production is limited in cultures by feedback inhibition. So, in situ adsorption of the compound should be preferred and also highly critical as a tool in bioprocessing for attaining the levels that are feasible for the industrial-scale production.

## 15. Immobilization, Combination of Elicitation, Immobilization and In Situ Adsorption of Plumbagin by Amberlite XAD-7 and Diaion^®^ HP-20, a Critical Tool for Enhancing Secondary Metabolite

Immobilization facilitates cell–cell contact by entrapping the cells in a polymer network. MS liquid medium containing 10 mM CaCl_2_ (devoid of sodium alginate) was used to give better strength to the beads [161]. Uncross-linked alginate also stimulated two times higher plumbagin synthesis in comparison with control cells. But, nearly 40% of the cells lost their viability in uncross-linked alginate [161], indicating that uncross-linked alginate if used results in a loss of cell viability. The immobilization of *P. rosea* cells in calcium alginate improved the plumbagin accumulation by 4.2-fold. Interestingly, immobilization also triggered 40% of extracellular product formation [161]. Such an extracellular product accumulation is ideal for upscaling the compound to an industrial level with the reutilization of cell biomass. They also have found the effect of cell density to the polymer concentration on plumbagin biosynthesis. Cell loading at a 20% level has been found quintessential for the accumulation of plumbagin but not at lower or higher concentrations [161]. These experiments indicate that the optimization of cell loading into the polymer is obligatory for effective product accumulation. When immobilization was coupled with elicitation using 200 mg/L chitosan, the accumulation of plumbagin was 36.17 mg/g dry cell wt. This plumbagin concentration is nearly 9- and 2-folds higher than the accumulation that was recorded in control cells (4.37 mg/g dry cell wt) and immobilized cells alone (16.14 mg/g dry cell wt), respectively, or slightly better than elicitation alone (28.94 mg/g dry cell wt) [164]. It may be noted that cells subjected to in situ extraction by Amberlite XAD-7 consumed more sucrose than the cells without it [161]. Permeabilization and in situ product adsorption led to superior yields of tanshinone and other bioactive molecules [253,256]. Nonionic polymeric ion-exchange resins such as Amberlite XAD series and also polystyrene resin have been used as adsorbents with excellent results. Heat shock coupled with ultrasound and in situ adsorption using styrene–divynilbenzene resin (Diaion^®^ HP-20) (Mitsubihsi Chemical Corporation, Tokyo, Japan) was performed in root cultures of *P. rosea* [246]. Heat stress at 60 °C alone for 10 min stimulated the plumbagin synthesis to 5.51 mg/g dry wt (5-folds). Contrary to this, no increase was recorded with ultrasound as an elicitor. On the other hand, after 60 °C of heat stress for 10 min in the presence of Diaion^®^ HP-20 (10 g/L), plumbagin content has boosted to 20.28 mg/g dry wt (14-folds) [246]. The addition of chitosan to 14-day-old root cultures of *P. indica* dramatically improved the plumbagin. But, the sequential addition of the resin Diaion^®^ HP-20 (Mitsubishi Chemical Corporation, Tokyo, Japan) after treating with chitosan enhanced the plumbagin content to 19.93 mg/g dry wt, which is 10-fold higher than the control root cultures of *P. indica* [248]. These experiments infer that the addition of resin is highly crucial for adsorption and an important strategy for us; thereby, plumbagin concentrations are elevated in cultured cells. Such strategies help us improve product recovery with relative ease and facilitate the successful commercialization of the products. The utilization of multiple adsorption resins with a synergistic effect in batch and semicontinuous cultures of plant cells might help to improve their product recovery and biosynthesis. Such a synergistic effect from three different resins produced acetylated taxanes in *Saccharomyces cervisiae* cultures [257]. But such a combination of resins has not been used so far in *P. rosea* cell cultures.

Wang et al. [258] noticed a six-fold increase in the paclitaxel accumulation in the suspension cultures of *Taxus chinensis* in aqueous-organic two-phase systems with the feeding of sucrose. Wang et al. [258] also reported a 63% release of the product into the medium. Hairy root cultures of *Salvia miltiorrhiza*, when grown in the presence of a resin, produced 7.4 times higher levels of the diterpenoid tanshinone with semicontinuous operation. This amount is higher in comparison with that of the batch culture [259]. Similarly, the removal of product using nonaromatic resin has led to an enhanced trans-resveratrol production in *Vitis vinifera* with salicylic acid and jasmonic acid elicitations [260]. Enhanced accumulation is mostly due to the use of adsorbent, since the product inhibition is controlled by harvesting the spent medium and the resin. Interestingly, synergistic effects of immobilization, elicitation and in situ product adsorption yielded 92.13 mg/g dry cell wt in suspensions of *P. rosea* in comparison with that of control cultures (4.37 mg/g dry cell wt) (Table 1). This is the highest amount of plumbagin that has been reported so far [161]. Thus, a synergistic effect has been noticed due to in situ quick product removal, which needs to be exploited further for the stabilized production of plumbagin in long-term commercial cultures. The cells do need not to be harvested for product extraction but can be reutilized from the spent medium.

## 16. Induction and Influence of Ammonium, Potassium, Calcium on Hairy Root Cultures of *P. rosea*

Hairy roots grow in basal medium without any hormones. Also, they are genetically stable. Hence, they are preferred for the production of secondary metabolites. Reports on hairy root cultures of *P. rosea* and *P. zeylanica* for plumbagin accumulation are scattered. Hairy root cultures were initiated from the in vitro grown shoots of *P. rosea* on MS basal medium by infecting them with *Agrobacterium rhizogenes* strain No. 15834 [205]. The doubling time of hairy roots was found to be 4.5 days. Hairy roots grew well on MS, Gamborg’s B5 [261], Schenk and Hildebrandt (SH) [262] and Linsmaier and Skoog’s (LS) [263] media but with different degrees of lateral branching. Of the four media tried for the plumbagin accumulation in *P. rosea* hairy root cultures, only MS medium exhibited 2.53 mg/g dry cell wt [205]. Hairy roots were initiated on MS medium containing 4% sucrose from *P. zeylanica* explants using the A4 strain of *A. rhizogenes*. Hairy roots accumulated 2.5 times more plumbagin than the control roots of the same age [208]. Hairy roots have been induced from several medicinally important plants like *Withania somnifera* [264], *Ferula pseudalliacea* [265] and *Scutellaria bornmuelleri* [266] for the production of bioactive compounds. Hairy roots of *P. rosea* displayed better growth and plumbagin accumulation in the pH range of 5.2 to 6.0. When the cultures were grown at a pH of 4.0 to 5.2, and also above pH 6.0, both the biomass and accumulation of plumbagin were repressed, and the hairy roots turned yellow and lost their viability [166,187]. Increasing concentrations of sucrose in the medium escalated the biomass to the 3% (*w*/*v*) level, but the content of plumbagin was enhanced to the 5% sucrose level and declined thereafter [194]. The hairy root growth of *P. rosea* and plumbagin accumulation were proportional to the initial concentrations (0.25×, 0.5×, 1×, 2× and 3×) of the KNO_3_, NH_4_NO_3_ and KH_2_PO_4_ supplemented to the medium [194]. Biomass and plumbagin were higher at normal levels of nitrate and ammonium, but higher concentrations suppressed their biomass and accumulation. K^+^ plays multiple physiological roles including abiotic stress tolerance in plants and perhaps can trigger secondary metabolism [267]. But the mechanistic explanation how KNO_3_, KH_2_PO_4_, and NH_4_NO_3_ trigger the plumbagin biosynthesis is largely elusive. Once they are metabolized, they may produce amino acids like tyrosine and alanine, which are the precursors of plumbagin. Therefore, it is reasonable to speculate that the nitrogen sources like nitrates could augment the plumbagin accumulation in cultures. Interestingly, when the CaCl_2_ concentration was three times more than the usual levels in the growth medium, the plumbagin content also augmented by 2-fold in comparison with the 1× level of CaCl_2_ [194]. CaCl_2_ also doubled the colchicine content in *Gloriosa superba* morphogenic cultures. Since calcium acts as a second messenger, it can trigger some of the downstream genes implicated in plumbagin synthesis [267]. Metals are also known to trigger the accumulation of secondary plant products in cultures [267]. Root and hairy root cultures were initiated and established from *P. zeylanica* using *Agrobacterium* strain MTCC532. The results indicate that MS medium was the ideal for hairy root growth rather than other media and sucrose as the best carbon source [268]. But they did not estimate plumbagin from these cultures. Half-strength MS-B5 liquid medium containing 30 g/L sucrose resulted in a good growth of hairy roots (1.8 g/dry cell wt) with a plumbagin accumulation of 3.2 mg/g dry cell wt from *P. zeylanica* [269].

## 17. Elicitation of Hairy Roots

High plumbagin content was noticed in methyl jasmonate-elicited hairy roots of *P. indica* [270]. Gangopadhyay et al. [158] also initiated hairy roots from *P. rosea*, which accumulated a maximum of 13.16 mg/g dry cell wt upon elicitation with chitosan and methyl jasmonate combination. *Agrobacterium rhizogenes* (strains A4, ATCC15834, MSU440, and A13)-mediated genetic transformation was carried out in *P. europea* leaf, hypocotyls and stem explants. From stem explants, hairy root induction with 69.3% frequency was noticed by using 100 µM acetosyringone. Adventitious and hairy roots have been initiated from *P. zeylanica* [198]. They recorded higher plumbagin content in hairy roots by incorporating an apocarotenoid elicitor α-ionone in the medium. The induction of hairy roots and accumulation of plumbagin in hairy roots is shown in Table 2.

## 18. Cell and Hairy Root Cultures of *P. rosea*, and Plumbagin Accumulation in Bioreactors

For the industrial-scale production of high-value compounds or pharmaceutically important compounds, suspensions need to be grown first in laboratory-scale or bench-scale model reactors. The viability of cells, as well as the stability and the suitability of cultures, can be verified for biomass production and secondary metabolite accumulation. But the type of bioreactor including its design, shape, volume, and cultivation regime greatly influences the synthesis and accumulation of bioactive compounds [174,275]. Reports on the culture of *P. rosea* and *P. zeylanica* cultures in bioreactors are sporadic, indicating the need to exploit this technology for the commercial production of plumbagin. *P. rosea* cell cultures were grown in different bioreactors like stirred tank, airlift, and tapered airlift reactors fabricated in the lab [194]. The growth of *P. rosea* cell cultures was compared to that of shake flask cultures, and the results ascertain that the growth of cells in bioreactors was slow and the exponential phase was prolonged to 24 days in contrast to 16 days in shake flask cultures. The growth of cell cultures was superior in the tapered airlift reactor (10.23 g dry cell wt/L) by 28 days of culture in comparison with the other two bioreactors mentioned above [194]. In all the reactors, an increase in cell growth was noticed with a concomitant increase in plumbagin accumulation. But the accumulation of plumbagin was the highest during the stationary phase of culture growth in *P. rosea* cell cultures. In the tapered airlift reactor, plumbagin accumulation was ~30 mg/L in comparison to that of stirred tank reactor grown cells (~15 mg/L) [194]. Thus, the accumulation of plumbagin in a tapered airlift reactor was seven times higher than free cells (nearly 4 mg/g dry cell wt) and two times higher than free cells with the chitosan elicitor (~15 mg/g dry cell wt) grown under identical conditions. The results indicate that the engineering considerations of a bioreactor such as thermodynamics, growth kinetics, etc., are vital for optimizing both growth and secondary plant product accumulation [174]. Gangopadhyay et al. [158] reported an induction of hairy roots from *P. rosea*. They treated hairy roots with a combination of elicitors such as 200 mg/L chitosan and 80 µm methyl jasmonate. This combination showed a synergistic effect on the accumulation of plumbagin (13.16 ± 1.72 mg/g dry cell wt) with the simultaneous leaching of plumbagin into the culture medium in the bioreactor. This amount was slightly higher than that noticed in hairy roots grown in a shake flask (11.96 mg/g dry wt). In a bioreactor, the hairy root biomass increase was 5 g/L, and the content of plumbagin was 1.425% [37]. They observed that inoculum density is the key to attaining optimum biomass and plumbagin levels in *P. rosea* hairy roots. Hairy root biomass enhanced 12-fold in 25 days, while the plumbagin was 1.5% higher in 25-day-old culture [196]. The above reports point out that there is an urgent need to augment our efforts to grow hairy roots in bioreactors and bring the product concentrations to a commercial level.

## 19. Semicontinuous Production of Plumbagin with Total Cell Recycle in a Bioreactor: An Alternative, Key Strategy

The growth kinetics of *P. rosea* cultures indicated that the accumulation of plumbagin was optimum during the stationary phase of growth. When the cell culture growth was minimal, conditions favored plumbagin accumulation [194]. Under normal conditions (devoid of any elicitors or permeabilizing agents), plumbagin accumulated in cells but was seldom released into the medium [199]. Long-term maintenance of the batch culture is impractical, because nutrients are depleted along with growth. An attempt was therefore made in *P. rosea* cell cultures to prolong the stationary phase of culture growth to enhance the volumetric productivity of the bioactive compound. A continuous culture process is usually used to maintain cell growth at steady-state levels. A continuous culture with a total cell recycle is an alternative strategy to maintain higher productivity on an industrial scale [194]. But the productivity of a continuous stirred tank reactor (CSTR) would be limited due to the loss of cells through the outlet stream. The condition where growth and production occur simultaneously should be preferred for higher rates of product accumulation in cells. On the other hand, if the production of bioactive compounds is non-growth-associated (product synthesis followed by a period of rapid cell growth), cultures must be maintained at a slow growing rate to retain their productivity. For the accumulation/release of secondary metabolites, batch cultures, fed-batch cultures, two-stage batch cultures and continuous chemostat cultures are generally used [276]. The strategies that are currently available for enhancing bioactive compounds in cell cultures include immobilization, elicitation and permeabilization with cell wall modifications, among others. Inducing cell wall modifications with chemicals (chitosan, ethylene, hydroxycinnamic acid, jasmonic acid, lasalocid sodium, lead and others), drugs (penicillin, cephalosporin), enzymes such as pectinase, pectate lyase, polygalacturonase or by making changes in boron and quasi-essential element silicon concentrations have proved successful [1,185,277,278]. But such methods are not being used frequently in plant cell cultures including *P. rosea* cultures. By effectively deploying these strategies, we can improve the accumulation of secondary products. *P. rosea* cells were immobilized and grown in a tapered airlift reactor [194], and the results indicate that ~70% of the plumbagin accumulated inside the cultured cells, while the rest comes out with the addition of 150 mg/L chitosan at an interval of 10 days. The medium was replaced with a new medium every 10 days, and cells were harvested after another 2-day culture period for measuring plumbagin [194]. The results point out that novel and innovative methods must be conceived for taking laboratory-scale bioreactor results to the industrial level.

## 20. Addition of Micronutrients, and Cell Wall Inhibitors for the Accumulation of Bioactive Compounds

In continuous cultures, it is advisable to add micronutrients into the medium at optimum concentrations at pertinent intervals of time. The addition of micronutrients like boron and silicon are known to affect the cell wall properties [279]. Boron is associated with cross-linking the side chains of rhamnogalacturonan II in pectins [280]. Boron deficiency can lead to the accumulation of phenolic compounds in tobacco [281] and olive leaves [282]. 

Silicon, on the other hand, stimulated the synthesis of artemisinin in *Artemisia annua* [283]. Therefore, the addition of boron and silicon singly or in combination in the semi-continuous or continuous culture medium at optimal levels will prime the secondary metabolism. These experiments have not been tried widely, and hence they are recommended for boosting the bioactive compound accumulation in vitro including *P. rosea* cell cultures. Cellulose biosynthesis inhibitors such as dichlobenil and isoxaben have been known to alter the cell wall composition [284,285]. Maize cells which are habituated to grow in the medium containing dichlobenil displayed differences in cell wall composition, thereby significantly enhancing cinnamic acid derivatives [286]. Cells habituated to cellulose biosynthesis inhibitors, if fed with precursors, can hike the accumulation of bioactive compounds. But, such an attempt has not been made in *P. rosea* callus, suspensions or hairy roots so far.

## 21. Tasks That Require Immediate Attention

There is every need to improve the accumulation of important bioactive compounds in bioreactors using novel and innovative techniques like sequential elicitations, cell line selections, cell aggregate size, percent cell loading into the matrix, the incorporation of cellulose biosynthesis inhibitors, in situ adsorptions using multiple resins, and others so as to take them to the commercial scale. Another critical aspect that needs the attention of plant biotechnologists is the glycosylation (sugar conjugation) of secondary metabolites, especially using bioreactors. Glycosylation is crucial, since it makes the bioactive molecules stable. Glycosylation is a major regulator of phenylpropanoid availability and biological activity and stability [185,287,288]. If glycosylated molecules are stored in the vacuoles [288,289], then methods can be evolved to secrete them into the spent medium. Few micro RNAs (miRNAs), ncRNAs (noncoding RNAs) and siRNAs (small interfering RNAs) have been discovered so far associated with the synthesis of anthocyanins, steroidal saponins, (S)-laudanosin, (S)-tetrahydrocolumbamine, and *Vinca* alkaloids [290,291], but not for plumbagin and other compounds. This necessitates discovering important miRNAs and their deployment, which can help to modulate the specific biosynthetic pathways that produce important bioactive compounds in culture for commercial production.

## 22. Conclusions

Plumbagin is an important anticancer molecule, but it is produced in low quantities in the native plants, mostly in the roots of *P. rosea*. Callus cultures, suspensions and hairy root cultures have been established both in *P. rosea* as well as in *P. zeylanica*. Regrettably, cell cultures or hairy roots produce inadequate quantities of plumbagin that are not feasible commercially. In view of its heavy demand in pharmaceutical industries, it is obligatory to upscale its production using cell and hairy root culture systems through a variety of techniques like elicitation, immobilization or a combination of them or sequential elicitations coupled with in situ adsorption using multiple resins as a tool in bioprocessing. So far, elicitation has been used singly but not in combination or sequentially with immobilization and in situ adsorption. However, the addition of micronutrients like boron and silicon and cell wall inhibitors can boost the buildup of bioactive compounds in culture, and these options needs to be explored widely. The loss of secondary metabolite accumulation over a period of time in culture is one of the bottlenecks which needs to be addressed forthwith. The selection of cell lines and glycosylation of compounds, often a neglected aspect, are vital for the production of high amounts of bioactive molecules on an industrial scale over a period of time without any loss of viability or decline in the concentration. Further, the multiple steps involved in the complex biosynthetic pathway and the corresponding genes that encode the proteins and epigenome that modulates the bioactive compounds in culture must be uncovered downright. An overexpression of the genes implicated in the pathway would certainly help produce plumbagin at an industrial scale.

## Figures and Tables

**Table 1:** Plumbagin accumulation in callus, and suspension cultures.

Species	Explant	Type of Culture	Media	Plumbagin Accumulation	Reference
*Plumbago zeylanicum*	Stem	Callus	-	Plumbagin accumulation depends on anthocyanin pigment	[200]
*Drosera rotundifolia* and *D. intermedia*	Whole plants	Whole plants grown in culture	-	Detection of plumbagin	[201]
*Drosophyllum lusitanicum*	-	Suspensions	MS medium	3.5% fresh wt	[189]
*Drosophyllum lusitanicum*	-	Suspensions	MS + chitin	Plumbagin released into the medium	[202]
*Drosera spathulata*, *D. rotundifolia*	-	In vitro cultured plants	-	Detection of plumbagin	[203]
*P. rosea*	Leaf	Cell aggregate size of 500 µm	MS + 1.5 mg/L IAA + 0.5 mg/L NAA + 0.3 mg/L BAP	1.83 mg/g dry wt	[190]
*P. rosea*	Leaf	Cell aggregate size of 1500 µm	MS + 1.5 mg/L IAA + 0.5 mg/L NAA + 0.3 mg/L BAP	4.57 mg/g dry wt	[190]
*Drosera binata*	Whole plantlets	Plant culture	MS medium + no hormones	1.4% dry wt	[204]
*Dionaea* *muscipula*	Whole plantlets	Plant culture	McCowns Woody Plant’s medium + no hormones	5.3% dry wt	[204]
*P. rosea*	Leaf	Suspensions	MS + 1 mg/L IAA + 0.3 mg/L BAP + 30 g/L glucose	4.06 mg/g dry wt	[194]
*P. rosea*	Leaf	Suspensions	MS + 1 mg/L IAA + 0.3 mg/L BAP + 30 g/L sucrose	3.85 mg/g dry wt	[194]
*P. rosea*	Leaf	Suspension-derived cell line PR10	MS + 1 mg/L IAA + 0.3 mg/L BAP	5.496 mg/g dry wt	190]
*P. rosea*	Leaf	Suspensions	MS + 1 mg/L IAA, 0.5 mg/L NAA, 0.3 mg/L BAP + 200 mg/L chitosan	28.94 mg/g dry wt (645% increase over that of control)	[205]
*P. rosea*	Stem, leaf	Callus and suspension cultures	MS + 1.5–2.5 mg/L 2,4-D + 0.5–1.5 mg/L KN	0.05 mg/g dry wt and 0.028 mg/g dry wt	[206]
*P. rosea*	-	Root cultures	Gamborg’s (B5) medium + 1 mg/L NAA + 0.1 ng/L kinetin	0.016% dry wt	[207]
*P. rosea*	Young leaf	Root cultures	Gamborg’s + 1 mg/L NAA + 0.1 mg/L kinetin	0.023% dry wt	[207]
*P. rosea*	Young leaf	Root cultures	Gamborg’s + 1 mg/L NAA + 0.1 mg/L kinetin + (NH_4_)_2_SO_4_	0.020% dry wt	[207]
*P. zeylanica*	Nodal explant	Axillary buds	MS + 8.87 mmol/L BAP + 0.49 mmol/L IBA	0.017% fresh wt	[208]
*P. rosea*	Leaf	Suspensions	MS + 1 mg/L, 0.5 mg/L NAA + 0.3 mg/L BAP	3.97 mg/g dry wt	[160]
*P. rosea*	Leaf	Suspensions	MS + 1 mg/L IAA + 0.3 mg/L BAP	4.92 mg/g dry wt	[160]
*P. roae*	Leaf	Suspensions	MS + 1 mg L/L IAA, 0.5 mg/L NAA, 0.3 mg/L BAP + Immobilization + Elicitation + In situ product removal by Amberlite XAD-7	92.13 mg/g dry wt	[161]
*Diospyros melanoxylon*	Leaf or petioles	Callus	MS + 2 mg/L 2,4-D + 1 mg/L BAP	2.2 mg/g dry wt	[63]
*Diospyros melanoxylon*	Leaf or petioles	Suspensions	MS + 2 mg/L NAA + 0.5 mg/L kinetin + 30 g/L sucrose + 100 µM jasmonic acid	3.1 mg/g dry wt	[63]
*Nepenthes khasiana*	Leaf, shoot tips, or roots	Callus	MS + 2 mg/L 2,4-D + 1 mg/L kinetin	1.8 mg/g dry wt	[68]
*N. khasiana*	Leaf, shoot tips, or roots	Suspensions	MS + 2 mg/L NAA + 1 mg/L BAP + 30 g/L sucrose + 100 µM jasmonic acid	3.4 mg/g dry wt	[68]
*Drosera indica*	Stem segments	Whole plant culture	¼ MS + 0.5 mg/L BA + 0.5 mg/mL yeast extract	2.69 mg/g dry wt (5.4-fold over the control plant)	[48]
*P. indica*	Young leaf	Root cultures	B5 + 0.1 mg/L NAA elicited by 20 Gy γ-ray irradiation	1.04 mg/g dry wt	[209]
*P. auriculata*	In vitro generated roots	Callus	MS + G2 media (0.2 mg/L BAP + 0.02 mg/L NAA)	0.35% dry wt	[210]
*P. auriculata*	Stem	Callus	MS + 1 mg/L 2,4-D + 1.75 mg/L NAA + 0.5 mg/L BAP + 1.5 mg/L NAA + 1 mg/L IAA	0.023% dry wt	[210]
*P. auriculata*	Leaf	Callus	MS + 1 mg/L 2,4-D + 1.5 mg/L NAA + 1 mg/L IBA	0.0145% dry wt	[210]
*P. rosea*	Leaf	Adventitious root cultures	MS + 1.5 mg/L IAA + 1 mg/L IBA + 50 μM jasmonic acid	1.23% dry wt	[195]
*P. zeylanica*	Leaf	Adventitious roots	MS solid medium + 1 mg/L IBA, 10 µM α-ionone	3.1 fold increase	[198]
*Drosera peltata* *D. burmannii*	Shoot clumps	Shoot culture	MS medium + 5 mg/L 2,4-D + 30 g/L sucrose	3.45 ± 0.90 mg/g dry wt	[211]
*P. zeylanica*	Nodal explants	Callus	MS medium + 5 μM IBA/TDZ/IAA + 100 mg/L yeast extract and 25 μM salicylic acid	0.55 mg/g dry wt with yeast extract and 0.32 mg/g dry wt with salicylic acid	[212]
*D. peltata*	Shoot tips	Shoot tip cultures	Half strength MS + 0.1 mg/L BA	12.04 mg/g dry wt from regenerated shoots	[211]
*P. zeylanica*	Nodal explants	Root cultures	Half strength liquid MS + 3% sucrose + 2 g/L inoculum density + 150 mg/L yeast extract	3-fold enhancement	[38]
*P. europaea*	Seeds, roots	Callus and suspensions	MS + 2,4-D + Kin/BA	0.9 mg/g dry wt	[38]
*Drosera indica*	Leaf, stem or root	Callus	MS + 5 mg/L 2,4-D + 1 mg/L BAP + 30 g/L sucrose	2.0 mg/g dry wt	[49]
*Drosera indica*	Leaf, stem or root	Suspensions	MS + 1 mg/L NAA + 0.5 mg/L BAP + 100 µM methyl jasmonate	4.0 mg/g dry wt	[49]
*Dionaea muscipula*	Leaf, petiole or root	Callus	MS + 2 mg/L 2,4-D + 1 mg/L BAP + 30 g/L sucrose	1.4 mg/g dry wt	[213]
*Dionaea muscipula*	Leaf, petiole or root	Suspensions	MS + 1 mg/L NAA + 0.5 mg/L BAP + 30 g/L sucrose + 100 µM methyl jasmonate	3.5 mg/g dry wt	[213]
*P. indica* *P. indica*	-	Regenerated shoots Regenerated shoots	MS + 1 mg/L BA + 50 mg/L yeast extract MS + 1 mg/L BA + 100 mg/L yeast extract	3.88% dry wt 3.81% dry wt	[214]

**Table 2:** Plumbagin accumulation in hairy root/teratoma cultures.

Species	Medium	Elicitor Used If Any	Strain	Plumbagin Accumulation	Reference
*P. rosea*	MS + 3% sucrose	Grown in a stirred tank reactor for 28 days	*Agrobacterium rhizogenes*	~12.5 mg/L	[194]
*P. rosea*	MS + 3% sucrose	Grown in a tapered airlift reactor for 28 days	*Agrobacterium rhizogenes*	~30 mg/L	[194]
*P. rosea*	MS + 3% sucrose	CaCl_2_ (0.25X–3X)	*Agrobacterium rhizogenes*	2.1–2.53 mg/g dry wt	[205]
*P. zeylanica*	Half-strength MS with 4% sucrose	-	*Agrobacterium rhizogenes* A4 strain	0.042% fresh wt 2.5 times higher amounts of plumbagin	[208]
*P. rosea*	Hormone-free liquid MS + 3% sucrose	-	*Agrobacterium rhizogenes* ATCC 15834	7.8 mg/g dry wt	[271]
*P. zeylanica*	MS basal without hormones	-	*Agrobacterium rhizogenes* MTCC 532	Not estimated	[268]
*P. rosea*	Hairy roots grown in half-strength MS medium	-	*Agrobacterium rhizogenes* strain ATCC 15834	Identified by TLC, but not estimated	[272]
*P. rosea*	Hairy roots grown on 0.5 mg/L GA_3_ + 0.5 mg/L NAA	-	Hairy root clone H13	7.90 mg/L dry wt	[158]
*P. rosea*	MS basal liquid in a bioreactor	-	Hairy roots	Plumbagin obtained in bioreactor as against 5.39-fold in shake flasks with 1% *w*/*v* inoculum over 3-weeks	[273]
*P. zeylanica*	MS medium free from hormones	-	*Agrobacterium rhizogenes* A4 and LBA9402 strains	A4 transformed HRA2B5 2.26 mg/g dry wt	[274]
*P. rosea*	MS basal in a bioreactor	-	*Agrobacterium rhizogenes* A4 (ATCC43057)	1.425% in a bioreactor	[37]
*P. rosea*	MS basal liquid in a 2L reaction kettle	Customized reaction kettle	*Agrobacterium rhizogenes* A4 strain	1.5% dry weight	[196]
*P. zeylanica*	MS + 1 mg/L IBA. Hairy roots grown in a bioreactor	10 µM α-ionone	*Agrobacterium rhizogenes* LBA1334, and R1000	3.6 fold increase in plumbagin in a bioreactor	[198]
*P. auriculata*	1/2 MS liquid medium	100 μmol/L methyl jasmonate	*Agrobacterium rhizogenes* (PAHR) 15834	8.24 mg/g dry wt at 25 days	[20]

## Data Availability

The data are available with the corresponding author K.K.B.P.
